# Pott's disease with extensive cold abscess in the abdominal cavity which was misinterpreted as malignancy

**DOI:** 10.1016/j.radcr.2022.01.070

**Published:** 2022-03-03

**Authors:** Deasy Kartika Peranginangin P, Widiana Ferriastuti

**Affiliations:** Department of Radiology, Faculty of medicine, Universitas Airlangga, Dr. Soetomo General Hospital, Surabaya, Indonesia

**Keywords:** Tuberculosis, Pott's disease, Cold abscess

## Abstract

Pott's disease is a distinctive presentation of tuberculosis that occurs in approximately 5% of extrapulmonary cases that progressively developed a voluminous paravertebral abscess. While the disease is marked only by the occurrence of inflammatory symptoms and low-grade pain, the advanced mimics other infections and malignancies. Therefore, early recognition is important for proper treatment preventing deformity of the residual spinal and permanent neurological deficit. We present a 20-years-old woman who experienced low back pain for 2 years and presented with a right-side lump in the abdomen. CT and MRI were performed in this case. CT image showed bone destruction and extensive abscess formation, while on an MRI there was epidural granulation and compacted cauda equina resulting in severe central canal stenosis. To clarify the diagnosis, a chest radiograph and Mantoux test were performed and the patient was confirmed positive for lung tuberculosis. After antituberculosis drug treatment, (Isoniazid, Rifampicin, Pyrazinamide, and Ethambutol) initial phase, the patient had difficulty walking.

## Introduction

Percival Pott presented the classical description of spinal tuberculosis (TB) in 1779, and spinal TB hence the name ‘Pott's Disease’ [1]. It occurs in approximately 1%-2% of all TB cases and is considered the most common and the most dangerous musculoskeletal manifestation of TB. Pott's disease occurs in about 50% of cases and is usually derived from an extra-spinal infection via a hematogenous route [2], [3]. Thereafter, Pott's disease may circulate to a juxtaposed intervertebral or vertebra disc. The spinal canal can be affected by granulomatous tissue or abscess due to direct spread from the vertebral lesion and results in decreased range of motion of the spinal cord, cord compression, and later neurological complications (eg, paresis and paraplegia) [3]. Therefore, early recognition and proper treatment are required to prevent deformity of the residual spinal and permanent neurological deficit. Current diagnosis by computed tomography (CT) and magnetic resonance imaging (MRI) is frequently carried out to evaluate spinal disorders [4]. We present the case of a 20-year-old woman whose diagnosis was confirmed as Pott's disease with extensive cold abscess. The patient was later on treated with an antituberculosis drug which contains Isoniazid, Rifampicin, Pyrazinamide, and Ethambutol.

## Case report

A 20-year-old woman presented with complaints of periodical pain in her lower back for 2 years and complained of a lump on the right side of her abdomen. She went to a neurologist and was treated with an analgesic. It turned out that the treatment was not reducing her pain so she willingly performed a massage of the area. Nevertheless, the lump's size increased and the patient had a fever for 5 days and shortness of breath.

**Fig. 1:**
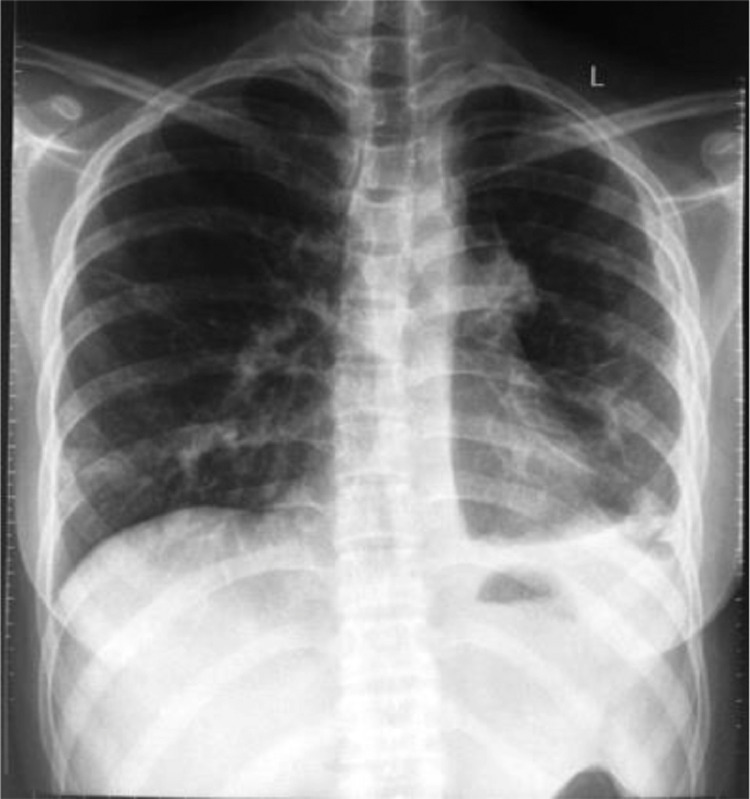
Chest radiograph showing minimal left-sided pleural effusion (star)

**Fig. 2:**
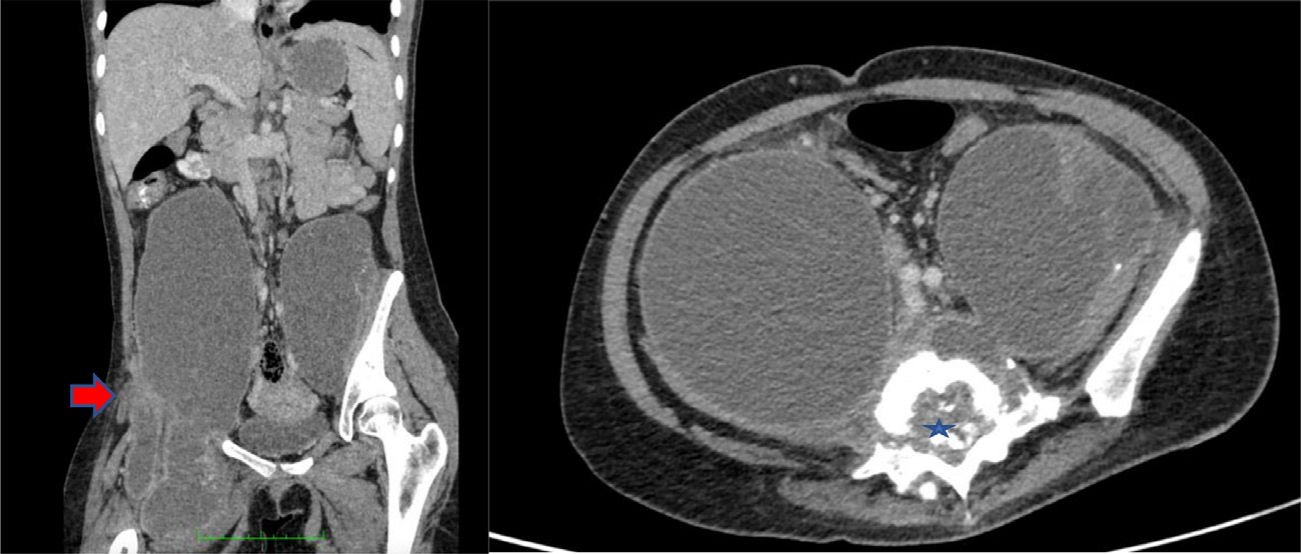
Contrast-enhanced abdominopelvic CT: (A) coronal plane demonstrated an extensive abscess in prevertebral space and revealed a psoas abscess in the right-left up to right inguinal fossa (red arrow); (B) the axial plane showed a retro vertebral abscess suggesting compression of the thecal sac (blue star)

**Fig. 3:**
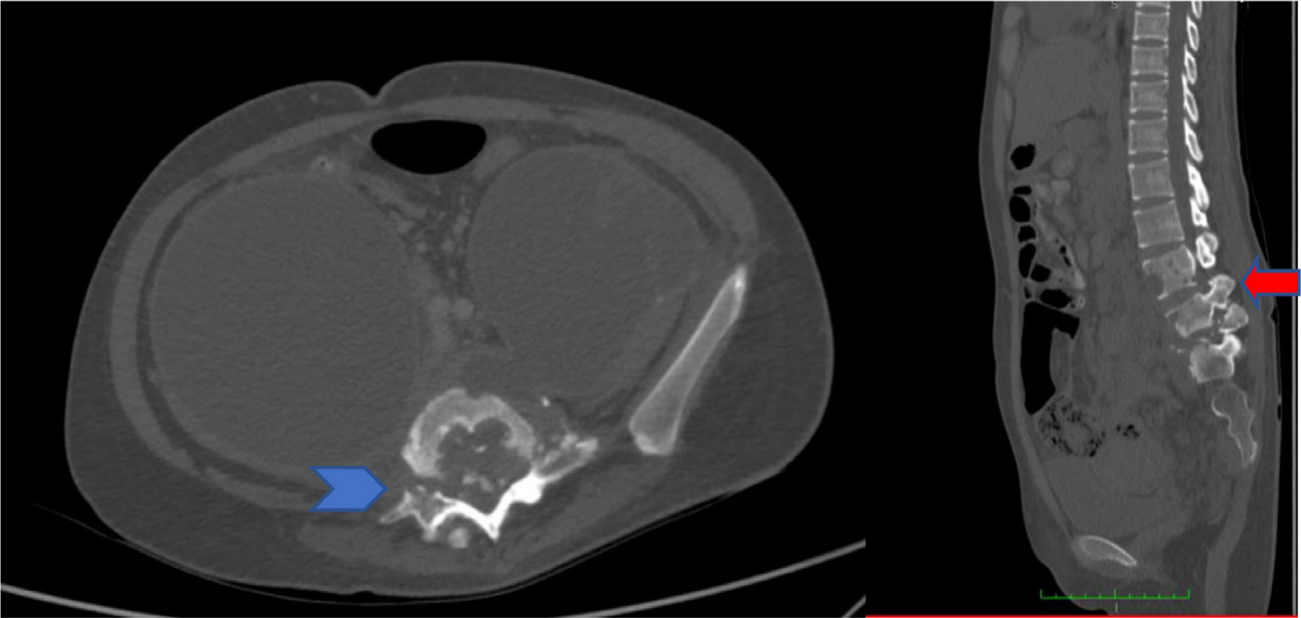
Abdominopelvic CT with bone window: (A) axial plane showed destruction of the L5 vertebra into the spinal cord with abscess formation in the prevertebral and paravertebral spaces, psoas abscess, and retro vertebral space (blue arrow); (B) the sagittal bone window showed bone destruction of the L4–5 vertebrae and S1 (red arrow)

Chest radiograph revealed a minimal left-sided pleural effusion (Fig.1). An abdominal ultrasound was carried out to visualize the abdominal lump, which resulted in the observation of a cyst in the right ovary which was suspected to be malignant. For this reason, she was referred to a gynecologist and was advised to undergo surgery. However, she refused and instead underwent herbal treatment. After 1 month of herbal treatment, her pain persisted and she had lost weight. At this time, she agreed to have surgery. An abdominopelvic CT scan was performed and revealed an extensive abscess in the prevertebral to retro vertebral spaces and a psoas abscess in the right-left up to right inguinal fossa (Fig. 2). Moreover, a bone window CT scan showed destruction of the vertebrae, including fourth until fifth lumbar, first sacrum, and the right and left fifth transverse processes (Fig. 3).

**Fig. 4:**
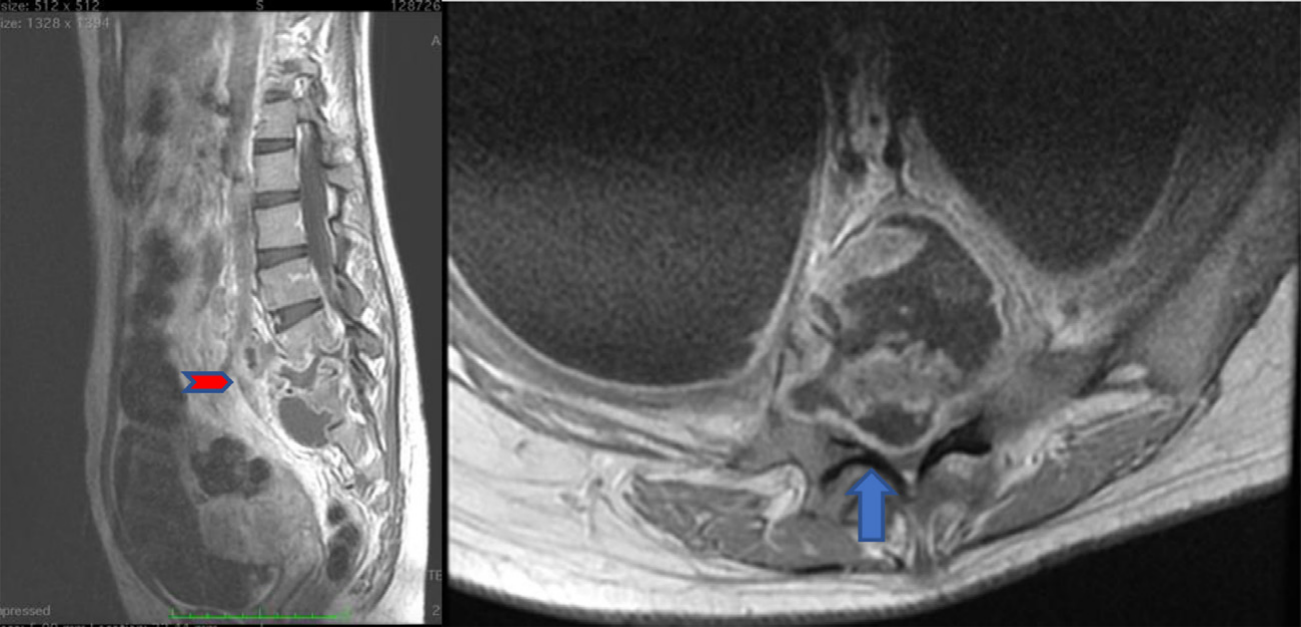
Contrast-enhanced MRI: (A) sagittal plane showed destruction of the discus and superior endplate corpus vertebra of L4 and L5 with an intraosseous abscess in the L4 and L5 corpus vertebral, expanding to the anterior subligamentous (red arrow); prevertebral abscess, right and left paravertebral abscess, and retro vertebral abscess; (B) the axial plane showed the retro vertebral abscess and granulomatous tissue in the thecal sac and compacted cauda equina resulting in severe central canal stenosis (blue arrow)

The patient started a treatment of antituberculosis drugs. However, she suddenly became unable to stand or walk. Because of this unexpected reaction, an MRI examination was performed. Enhanced MRI showed destruction of the disc and inferior endplate of the fourth lumbar vertebra , superior endplate of the fifth lumbar vertebra, superior endplate fith lumbar vertebrae which expanded to the anterior subligamentous, the right and left paravertebral abscess, also retro vertebral abscess urge the thecal sac, with epidural granulation and compacted cauda equina resulting in severe central canal stenosis (Fig. 4).

## Discussion

Pott's disease is the term for the destruction of the vertebral body and intervertebral disc caused by *Mycobacterium tuberculosis* (MTB) and is also known as tuberculous spondylitis [2]. The preferred sites which the infection expands are the vertebra thoracic segments followed by lumbar segments. Pott's disease often affects 2 contiguous vertebral bodies with the intervening disc; however, multilevel extension (3 or more vertebrae) is uncommon [6]. The involvement of single or multiple vertebral discs has been observed and remains unclear whether it was caused by the hematogenous dissemination. Other than the multiple involvements of the vertebra, multiple complaints were might be caused by direct subligamentous, paraspinal, or subarachnoid spread. Of these, the direct subligamentous spread seems most common. In our case, 2 segments of the lumbar vertebrae were affected by the intervening disc.

The normal route of entry for MTB is from the respiratory tract with hematological spread to the target site. Moreover, secondary hematological seeding can occur from a silent focus in other parts of the body, for example, the Genito-urinary tract, gut, or tonsils. Another route of spread to vertebral bodies is through the lymphatic system, which originates from contiguous para-aortic lymph nodes. Typically, more than 1 vertebra and more than 1 component of the spine are involved (ie, the vertebral body, intervertebral disc, ligaments, para-vertebral soft tissues, and the epidural space). The process begins with the damage of the cancellous bone which extends to the cortex. The inflammation slowly spreads to the vertebrae through the disc space or sub-ligament. In advanced disease, there is a progressive vertebral collapse resulting in kyphosis and gibbus formation. The spinal cord may become compressed by bony parts and/or the expanding abscess. Moreover, direct involvement of the cord and leptomeninges by granulation tissue is also observed. The involvement of these spinal structures in the patient resulted in cervical cord compression from the epidural collection and vertebral body retropulsion [2]. In this case, it was found that there was the involvement of the anterior subligamentous and cold abscess that extended from the retro vertebral space to the epidural space, causing compaction of the cauda equina.

Because the clinical presentation is indistinct and nonspecific, a definitive diagnosis is often difficult to make [4]. The pain overlying the affected vertebrae, low-grade fever, chills, weight loss, and nonspecific symptoms are likely to occur in most cases. Weakness and pain in the back and lower limb have been reported as the most common features in some local studies [2], [5]. In this study, the patient had back pain for 2 years and a lump in the right abdomen which was suspected to be a malignancy. The delay in diagnosis caused the patient to develop instability in her lower limbs. After 2 months was treated with antituberculosis drugs which in her regaining her ability to walk.

[1], [2], [3], [4], [5], [6] MRI is a suitable examination in terms of excellent visualization of the bone and soft tissue components of spinal TB. The imaging modality also identifies the disease at distant, asymptomatic sites. Even though CT is useful for assessing bone destruction, it is less accurate for visualizing the epidural extension of the disease. Nevertheless, it affects neural structures. MRI revealed soft tissue disease and its effect on the theca, cord, and foramen; this could not be confirmed with the CT findings [4].We performed CT and MRI examinations to identify the destruction of the affected disc and body as well as the surrounding soft tissue. We found that there was the destruction of the disc in the fourth and fifth lumbar vertebral bodies involving the anterior subligamentous and right and left psoas muscles, the presence of epidural granulation, and a retro vertebral abscess compressing the thecal sac and causing severe central canal stenosis at this level.

## Conclusion

Pott's disease is the most dangerous form of musculoskeletal tuberculosis because it can cause bone destruction, deformity, and paraplegia. Early diagnoses are important to prevent permanent disability. Imaging studies such as MRI and CT are important examination for characterizing the lesion, performing the biopsy, planning surgery, evaluating the success of treatment, and detecting complications in the follow-up.

## Patient consent

Written informed consent was obtained from the patient for the publication of this case report.

## References

[1] Ansari S, Amanulah F, Kaleem A, Raj K. (2013). Pott's spine: diagnosis imaging modalities and technology advancements. North Am J Me Sci.

[2] Okwudire E.G. (2021). Atypical presentation of cervical pott's disease: a case report. J Tuberculos Res.

[3] Singh KS, Kurian MM, Babu AR, Rajan NR, Sherief SH. (2019). A Case report on pott's spine (Spinal Tuberculosis). IJRR.

[4] Sinan T, Al-Khawari H, Ismail M, Ben-KA Sheik M.CT, Feature MRI (2004). Ann Saudi Med.

[5] Garcia-Rivas A, Estrada-SS Odin TC, Gomilla CL, Elisa Franquet (2012). Eur Spine J.

[6] Kumar Garg R., Somvanshi Dilip Singh (2011). Spinal Tuberculosis : A review. The Journal of Spine Cord Medicine.

